# Cognitive flexibility, central coherence, and quality of life in anorexia nervosa

**DOI:** 10.1186/s40337-022-00547-4

**Published:** 2022-02-15

**Authors:** Timo Brockmeyer, Hagen Febry, Anna Leiteritz-Rausch, Wally Wünsch-Leiteritz, Andreas Leiteritz, Hans-Christoph Friederich

**Affiliations:** 1 Department of Clinical Psychology and Psychotherapy, Institute of Psychology, University of Goettingen, Gosslerstrasse 14, 37073 Goettingen, Germany; 2 Department of General Internal Medicine and Psychosomatics, Center for Psychosocial Medicine, University Hospital Heidelberg, Im Neuenheimer Feld 410, 69120 Heidelberg, Germany; 3 Klinik Lueneburger Heide, Am Klaubusch 21, 29549 Bad Bevensen, Germany

**Keywords:** Set-shifting, Global/local processing, Cognitive functions, Neuropsychological functions, Cognitive control, Daily functioning, Eating disorders

## Abstract

**Background:**

Anorexia nervosa (AN) has consistently been found to be associated with poor cognitive flexibility and central coherence. These two cognitive functions have been considered important maintenance factors in AN and are addressed by specific treatment approaches such as cognitive remediation therapy. While there is clear empirical evidence that difficulties in such cognitive functions are related to impaired daily functioning in schizophrenia and bipolar disorder, this potential association has received only little attention in AN research so far. Therefore, the aim of this study was to examine potential relationships between weak cognitive flexibility, central coherence, and poor quality of life (QoL) in AN.

**Methods:**

Cognitive flexibility and central coherence were measured by both neuropsychological (i.e., performance based) and self-report measures alongside with self-reported QoL in a sample of 138 adult patients with AN.

**Results:**

Self-report but not performance based measures of cognitive flexibility and central coherence were associated with QoL. Weaker cognitive flexibility and central coherence were correlated with poorer QoL. These associations were independent of comorbid depression. The link between weak central coherence and poor QoL was particularly strong in patients with the restricting subtype of AN. The link between cognitive flexibility and QoL, however, was independent of AN subtype.

**Conclusions:**

Weak cognitive flexibility and central coherence are associated with low QoL in AN, especially in patients with the restrictive subtype. However, this relationship is dependent on the method of measurement, where self-report measures seem to be more relevant than performance based measures.

## Plain English summary

Cognitive flexibility is a mental function that refers to the ability to switch flexibly between different rules, tasks, and actions. Central coherence is another mental function that is often considered as 'bigger picture' thinking or the ability to see 'the forest for the trees', whereas weak central coherence is mirrored by an excessive focus on detail. Previous research has shown that anorexia nervosa (AN) is associated with both weak cognitive flexibility and weak central coherence. These two factors have been incorporated in theoretical models of the development and maintenance of AN and are targeted in specific treatments for AN. It is known that these two factors are associated with poor quality of life (QoL) in other mental disorders such as schizophrenia. However, only little attention has been paid to the question whether they are also associated with QoL in AN. In the present study, neuropsychological (performance based) and self-report measures were used to assess cognitive flexibility and central coherence alongside QoL in 138 adult patients with AN. We found that only low self-reported levels of cognitive flexibility and central coherence were associated with poor QoL. The association between central coherence and QoL was particularly strong in those patients who had the restricting subtype of AN.

## Background

Anorexia nervosa (AN) has consistently been found to be associated with weak set-shifting [1, 2] and central coherence [3]. Set-shifting is also referred to as cognitive flexibility and is the ability to adapt to changing environmental demands by flexibly switching between different rules, tasks, actions, and mental sets [4, 5]. Accordingly, weak set-shifting or cognitive inflexibility results in rigid or perseverative thinking and behaviour. Central coherence describes the ability to overview complex stimuli or information, and is often considered as 'bigger picture' thinking or the capability to see 'the forest for the trees', whereas weak central coherence is reflected by an excessive focus on detail [6]. Weak set-shifting was also observed in women recovered from AN and in unaffected first-degree relatives of patients with AN and has thus been considered a potential endophenotype [7, 8]. Both weak set-shifting and central coherence are typically mirrored by obsessive pre-occupations with food, body shape and weight, and compulsive calorie-counting and exercising in AN. As a result, they have been incorporated in maintenance models of AN [9, 10] and are addressed by specific treatment methods such as cognitive remediation therapy [11, 12] and the Maudsley Model of Anorexia Nervosa Treatment for Adults (MANTRA) [13, 14]. From research on other mental disorders, it is known that difficulties in these cognitive functions are associated with reduced daily functioning and quality of life (QoL) in schizophrenia [15] and bipolar disorder [16], and that cognitive remediation therapy can help to improve cognitive and daily functioning in patients with these disorders [17, 18]. It is also known that AN is associated with severe impairment in social and occupational functions and poor health-related quality of life (QoL) [19–22] and that poor psychosocial functioning and QoL predict future AN symptom severity and mortality [23, 24].

However, a recent systematic review has indicated that only very few studies to date have investigated whether and to what extent weak cognitive flexibility and central coherence are associated with impaired daily functioning or QoL in AN [25]. In two of these studies, cognitive flexibility was assessed by performance based tests such as the Wisconsin Card Sorting Test (WCST) and the Trail Making Test (TMT), and was not associated with functional impairment [26, 27]. One study found a significant correlation between the parameter 'difficulty maintaining set' in the WCST and QoL in AN [28]. However, this parameter is not considered to be a good indicator of set-shifting ability. It refers to the number of times the participant shows an incorrect response after a series of consecutive correct responses and may thus be more indicative of distractibility [29]. Another study found a correlation between 'categories completed' in the WCST and financial QoL in AN [30]. Again, however, 'categories completed' is not considered a good indicator of set-shifting ability but rather of general abstract reasoning or the ability to learn the rules of the task [25].

In sum, no relationship could be observed between established, performance based indicators of cognitive flexibility/central coherence and daily functioning/QoL in AN [26–28, 30]. However, sample sizes in these studies were mostly small and only performance based measures of cognitive flexibility and central coherence were used. Notably, previous studies indicate that performance based and self-report measures may map different aspects of cognitive flexibility and central coherence [31–34]. While performance based measures are considered to provide a relatively objective measure of an actual cognitive function in a single situation under almost ideal conditions, self-report measures are considered to map a summary of subjective experiences across different situations in daily life and possibly also metacognitions, but are also more susceptible to bias (e.g. through restrospection bias, reduced introspection, social desirability, aggravation or dissimulation).

Against this background, the aim of the current study was to investigate potential associations between cognitive flexibility, central coherence and quality of life using both performance based and self-report measures in a large, heterogeneous AN sample. Both for models of the development and maintenance of the disorder and for the development/ refinement and application of specific interventions, it seems important to better understand the relevance of reduced cognitive flexibility and central coherence for the daily functioning and quality of life in patients with AN. Because previous studies failed to establish a link between performance based measures of cognitive flexibility/central coherence and daily functioning/QoL and because of the common finding that associations between performance based and self-report measures are generally often low [31, 34], we expected that rather low self-reported than poor performance based cognitive flexibility and central coherence would be associated with poor QoL in AN. Since it has been discussed in the literature that both AN subtypes and comorbid depression may have an influence on cognitive flexibility and central coherence in AN, but the findings on this are inconsistent [35–39], we considered both factors as potential moderator variables in the current study, but without formulating a directed hypothesis on this in advance.

## Methods

### Participants

The total sample was comprised of 138 adult inpatients with AN. Participants were recruited consecutively from a specialised eating disorders clinic and assessed during the first ten days after admission. For being eligible to take part in the study, participants had to be at least 18 years old and had to meet AN criteria according to the DSM-5 [40]. Study assessors (trained psychologists and physicians with long-standing clinical experience) screened participants for eligibility using the Structured Clinical Interview for DSM disorders [41]. Comorbid depression and AN subtype were diagnosed in the same way. Exclusion criteria were an acute life-threatening condition, medical instability, or severe psychiatric comorbidity (i.e., schizophrenia, bipolar disorder). All participants provided written informed consent. The study was approved by the Ethics Committee of the Medical Faculty of the University of Heidelberg (S-031/2015), and the Ethics Committee of the Medical Association of Lower Saxony, Germany (Bo/279/2014). A power analysis yielded that, with this sample size, a small to medium effect (*r* = 0.24) could be detected in a bivariate correlation with α = 0.05 and power = 80%.

### Measures

#### Set-shifting

Set-shifting ability was measured using the *Wisconsin Card Sorting Test* (WCST) [42] and the *Trail Making Test* (TMT) [43]. These are the two most widely used measures of set-shifting in AN research and it has been shown in several meta-analyses that these measures are suitable for mapping weak set-shifting in AN patients [2, 44, 45]. In the WCST, participants have to match stimulus cards with one of four category cards. The sorting rule can refer to the colour, shape or number of displayed symbols. The sorting rules change several times during the task without being predictable. The participants have to figure out what the new rule is based on error feedback. Perseveration errors are the most commonly used and most sensitive performance index of set-shifting in the WCST. This index is determined by counting how often a participant sorts the cards according to a previously correct but now incorrect rule, despite negative feedback.

In the TMT, participants first have to connect numbered circles in the correct numerical order (i.e. 1–2–3; trail A). In the next run, however, they are asked to alternatively link numbers and letters (i.e. 1–A–2–B–3–C; trail B). Set-shifting performance can be indexed by the time taken to complete this second trail correctly. Both the WCST and TMT were performed using Inquisit 4 (Millisecond Software).

#### Central coherence

Central coherence was assessed by the *Navon Task* [46]. In this task, subjects are presented with large letters (e.g. H or S = global stimulus) formed from small letters (e.g. _H_ or _S_ = local stimulus). In congruent runs, the large letter (global stimulus) corresponds to the small letters (local stimuli) from which it is formed (e.g. a large H consists of many small _H_). In incongruent runs, the large letter (global stimulus) does not correspond to the small letters (local stimuli) of which it is made (e.g., here a large H is made of many small _S_). In one condition, participants are asked to respond to the global stimulus (e.g. they should press the H key when shown a large H, regardless of which small letters it is made of). In the other condition, they should respond to the local stimuli (e.g. they should press the H key when the small letters are _H_, regardless of what the large letter is). Longer reaction times in incongruent global runs may serve as a simple indicator of weak central coherence (since it can be assumed that with longer reaction times the processing of the global stimulus is more disturbed by incongruent local stimuli). In addition, two further indices can be calculated. One is the global interference effect. For this, the mean reaction times in congruent local runs is subtracted from the mean reaction times in incongruent local runs. This index reflects the extent to which the processing of local stimuli slows down when there is interference from an incongruent global stimulus. Low values in this index reflect a strong detail focus or advanced local information processing. The final index, the local interference effect, is calculated by subtracting mean reaction times in congruent global runs from mean reaction times in incongruent global runs. This index reflects the extent to which the processing of global stimuli is slowed down by interference with incongruent local stimuli. Probably, this is the most relevant indicator of weak central coherence. High values here indicate weak central coherence, i.e. greater difficulty seeing the 'bigger picture' (the global stimulus) when there is interference with incongruent local stimuli than when there is no such interference. The logic behind this is that if a subject focuses very strongly on details and these details are incongruent with the global stimulus, the processing of the global stimulus is more strongly compromised. This task has been used less frequently than others to investigate central coherence in AN but it has, nonetheless, been demonstrated to map weak central coherence in this population [47]. The advantages of this task, however, are that interference between local and global information processing can be mapped more directly than in other tasks and that potential measurement biases are reduced because a computerised version of the task is available. The task was performed using Inquisit 4 (Millisecond Software).

#### Cognitive rigidity and attention to detail

The *Detail and Flexibility Questionnaire* (DFlex) [48] was developed as a self-report measure of difficulty with set-shifting/cognitive flexibility and weak central coherence [48]. It is composed of two subscales with 12 items each: cognitive rigidity (mapping weak set-shifting/cognitive flexibility; e.g. 'I like doing things in a particular order or routine') and attention to detail (mapping weak central coherence; e.g. ' I can get lost in details and forget the real purpose of a task'). The developers of the questionnaire recommended to use the two subscales independently [48]. Answers are scored on a six-point Likert scale. Higher scores indicate more cognitive rigidity and more attention to detail, respectively. The scale has shown good to excellent reliability and validity in previous research and discriminated between patients with an eating disorder and healthy controls [48].

#### Health-related quality of life

Health-related quality of life (QoL) was assessed by the *Essen Quality of Life-Index for Eating Disorders* (ELI) [49]. This self-report questionnaire features excellent reliability and high validity [49]. It consists of ten items assessing mental, physical, and social impairments during the last four weeks. Answers are scored on a four-point Likert scale, with higher scores reflecting greater impairment.

### Statistical analyses

All analyses were performed using IBM SPSS Statistics version 25 (SPSS Inc., IBM Corporation, Armonk, New York, NY, USA). Univariate outliers (> 3 SD) were winsorised. Since QoL, WCST, and TMT data were not normally distributed, Spearman rank correlation coefficients were calculated to uncover potential associations between cognitive flexibility, central coherence, and QoL. Bootstrapping for Pearson Product Moment correlation coefficients was also performed with bias corrected and accelerated 95% confidence intervals and 10,000 bootstrap samples. However, this yielded the same results like the non-parametric Spearman Rank correlations. For ease of interpretation, we thus only report the latter. In addition, we conducted separate moderator analyses to examine potential effects of comorbid depression and AN subtype on the aforementioned associations. To this end, we used the PROCESS macro for SPSS [50]. Statistical significance was defined at the 0.05 *p*-level, two-tailed.

## Results

Demographic and clinical characteristics of the sample are summarised in Table 1. As can be seen, almost all participants were women, and the sample covered a broad age range (18–54 years) and also a broad range of symptom severity (as indicated by the body mass index ranging between 9.70 and 18.01). More than two thirds of the patients had the restricting subtype of AN.

**Table 1:** Demographic and clinical characteristics of sample (*n* = 138) and cognitive flexibility, central coherence, and quality of life data

	*M* (*SD*) or % (*n*)
Age (years)	25.32 (7.93)
Gender (% women)	98 (135)
Body mass index (kg/m2)	14.42 (1.83)
EDE-Q	3.69 (1.44)
AN subtype (% restricting)	72 (99)
WCST nr. of perserative errors	7.18 (3.99)
TMT trial B (seconds)	83.55 (42.42)
NT_incongruent global_	607.46 (500.40)
NT_global interference_	43.12 (128.46)
NT_local interference_	61.92 (284.30)
DFlex_cognitive rigidity_	43.96 (10.34)
DFlex_attention to detail_	49.09 (9.93)
ELI	25.10 (7.76)

AN = Anorexia Nervosa; EDE-Q = Eating Disorder Examination Questionnaire, EDE-Q data were available from 134 patients; WCST = Wisconsin Card Sorting Test (number of perseverative errors), WCST data were available from 119 patients; TMT = Trail Making Test (time for trail B), TMT data were available from 137 patients; NT = Navon Task, NT data were available from 108 patients; DFlex = Detail and Flexibility Questionnaire; ELI = Essen Quality of Life-Index for Eating Disorders

Cognitive flexibility, central coherence, and QoL data are also summarised in Table 1. Correlation coefficients together with *p*-values are displayed in Table 2. Performance based measures of cognitive flexibility (i.e., WCST, TMT) and central coherence (NT) were not correlated with QoL. However, greater self-reported attention to detail and cognitive rigidity were associated with lower QoL with a moderate effect size. Besides, self-report and performance based measures of cognitive flexibility and central coherence did not correlate with each other, and performance based measures only inconsistently correlated across each other.

**Table 2:** Correlations between cognitive flexibility, central coherence, and quality of life measures

	*ρ* (*p*)	*ρ* (*p*)	*ρ* (*p*)	*ρ* (*p*)	*ρ* (*p*)	*ρ* (*p*)	*ρ* (*p*)	*ρ* (*p*)
	ELI	WCST	TMT	NT _incongruent global_	NT _global interference_	NT _local interference_	DFlex _cognitive rigidity_	DFlex _attention to detail_
ELI	–							
WCST	0.15 (0.105)	–						
TMT	0.05 (0.532)	− 0.15 (0.097)	–					
NT_incongruent global_	− 0.03 (0.794)	− 0.33 (0.001)	0.46 (< 0.001)	–				
NT_global interference_	− 0.08 (0.422)	− 0.01 (0.944)	− 0.19 (0.047)	− 0.11 (0.248)	–			
NT_local interference_	0.02 (0.819)	− 0.16 (0.133)	0.14 (0.143)	0.36 (< 0.001)	0.04 (0.699)	–		
DFlex_cognitive rigidity_	0.28 (0.001)	0.13 (0.175)	0.13 (0.175)	− 0.07 (0.481)	− 0.17 (0.086)	0.10 (0.330)	–	
DFlex_attention to detail_	0.44 (< 0.001)	0.03 (0.750)	0.03 (0.750)	0.01 (0.938)	− 0.11 (0.280)	− 0.04 (0.671)	0.66 (< 0.001)	–

ELI = Essen Quality of Life-Index for Eating Disorders; WCST = Wisconsin card sorting test (number of perseverative errors); TMT = Trail Making Test (time for trail B); NT = Navon Task; DFlex = Detail and Flexibility Questionnaire

**Fig. 1:**
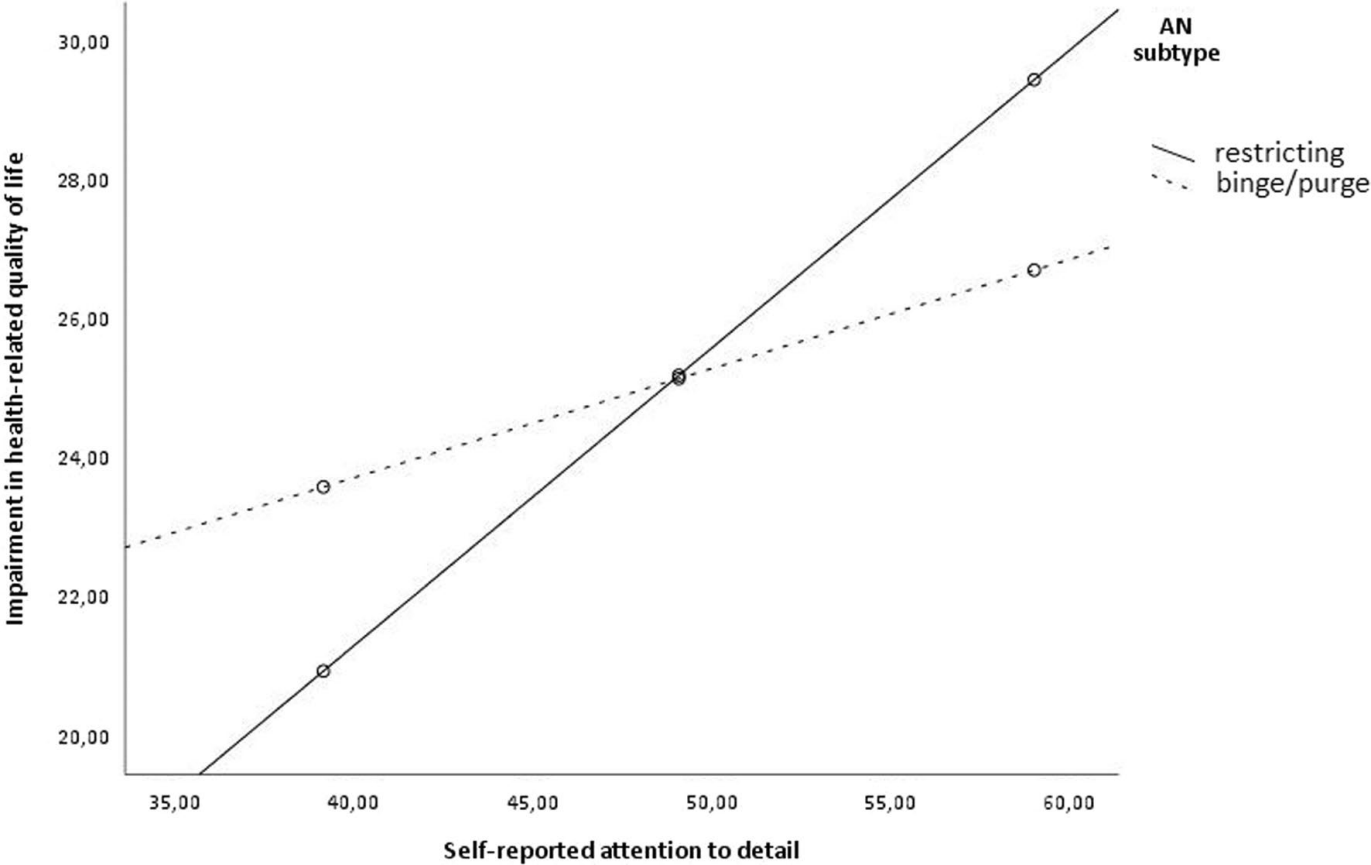
AN subtype as moderator of the association between attention to detail and quality of life

Additional moderator analyses revealed that AN subtype was a significant moderator of the association between self-reported attention to detail and QoL, Δ*R*^2^ = 0.03, *F*(1, 134) = 4.30, *p* = 0.040. Only in patients with the restricting subtype of AN, attention to detail was significantly correlated with QoL, *b* = 0.43, 95% bias corrected CI (0.289; 0.568), *p* < 0.001. In patients with the binge/purge subtype of AN, there was no such association, *b* = 0.16, 95% bias corrected CI (− 0.061; 0.375), *p* = 0.157. This moderator effect is illustrated in Fig. 1. However, AN subtype did not moderate the association between self-reported cognitive rigidity and QoL, Δ*R*^2^ = 0.02, *F*(1, 134) = 3.17, *p* = 0.077. Furthermore, comorbid depression did not moderate the associations between self-reported cognitive rigidity, attention to detail, and QoL (*p* = 0.176 and *p* = 0.472, respectively).

## Discussion

This is the first study examining potential associations between QoL and both neuropsychological (i.e., performance based) and self-report measures of cognitive flexibility and central coherence in AN. Consistent with our hypothesis, we found that self-report but not performance based measures of cognitive flexibility and central coherence were associated with QoL. Both high cognitive rigidity and attention to detail, as measured by the DFlex, were associated with lower QoL. This finding is novel. The finding also supports the call for more ecologically valid measures of cognitive functioning in AN [34, 51] because unlike performance based measures, self-report measures are considered to more closely reflect experiences in real-world settings. The current finding that the association between attention to detail and QoL is particularly strong in patients with the restrictive subtype of AN tends to be consistent with tentative evidence that this subtype is associated with weaker central coherence [38]. However, this result must be interpreted with caution since the moderator effect was only small and 72% of the participants had the restricting subtype. It could thus be that this simply reflects that the association between attention to detail and QoL is more consolidated in this larger subsample. The current finding that the aforementioned association between attention to detail and QoL is independent of comorbid depression tends to be in line with a recent systematic review that concluded that central coherence in AN is not affected by comorbid depression [37]. The finding that performance based measures of cognitive flexibility and central coherence are not related to QoL in AN is consistent with the findings of previous studies in smaller samples [26–28, 30]. In addition, the finding that self-report and performance based measures of cognitive flexibility and central coherence do not correlate is in line with earlier research in AN and in other domains [31, 32].While performance based measures are considered to provide a relatively objective depiction of actual cognitive function in a given situation under almost ideal conditions (i.e., in the lab), self-report measures are considered to provide information about cumulative subjective experiences across different real-world situations and possibly also metacognitions, but are also more susceptible to bias.

Although, of course, no causal conclusions can be drawn from correlational evidence, the present study adds to the understanding of the potential impact of weak cognitive flexibility and central coherence on daily functioning in AN. This is important since improvements in daily functioning are milestones of recovery and cognitive flexibility and central coherence have become treatment targets in AN [11, 13, 14] although until now it had been largely unclear whether these factors have any influence at all on daily functioning in AN. Future research may use prospective longitudinal designs to investigate whether weak cognitive flexibility and central coherence actually precede limitations in daily functioning in AN. Moreover, it would be particularly important to determine whether improvements in cognitive flexibility and central coherence during treatment predict improvements in daily functioning in AN. If this were the case, existing interventions could be adapted to focus more on these cognitive functions. In the light of the findings of the present study, it would be advisable in such future studies to use self-report measures or, for example, ecological momentary assessment [52–54] in addition to objective perfomance based measures of cognitive flexibility and central coherence in a single situation in the lab.

Strengths of the current study include the large, heterogeneous sample, the use of the two most established neuropsychological measures and the most established self-assessment measure of cognitive flexibility and central coherence in AN research [2, 25], and the inclusion of comorbid depression and AN subtype as potential moderator variables. However, the study also has some limitations. The cross-sectional design precludes any causal interpretations. Although it seems more plausible that weak cognitive flexibility and central coherence contribute to reduced QoL, it cannot be ruled out that the reverse is not true or that other, latent variables influence both. More prospective, longitudinal studies in even larger samples are needed to better understand potential causal relationships. Although the sample size of the present study was significantly larger than that of other studies in this realm in the past [26–28, 30, 55], it was still too small to detect small effects. Thus, it cannot be ruled out that with an even larger sample size, small sized correlations between WCST, TMT, and NT scores and QoL would be detected. As all participants in the current study were adult inpatients, and the vast majority female, the results may not be readily transferable to other patient groups (i.e., younger, male, and outpatients). Finally, the lack of a complete structured clinical interview and a measure for depression severity is another limitation of the current study.

## Conclusions

This study contributes to a better understanding of the relevance of weak cognitive flexibility and central coherence in patients with AN. The results indicate that weak self-reported cognitive flexibility and central coherence are associated with low QoL in AN. Neuropsychological measures, on the other hand, showed no association with QoL. The associations between poor cognitive flexibility, central coherence, and QoL were independent of comorbid depression. The association between attention to detail and QoL, however, was significantly stronger in patients with the restricting subtype of AN whereas the link between cognitive rigidity and QoL was independent of the AN subtype. These findings provide tentative support for the assumption that self-assessed cognitive flexibility and central coherence are relevant treatment targets in AN. The current findings furthermore suggest that self-report measures are used in addition to objective perfomance-based measures of cognitive flexibility and central coherence in AN research.

## Data Availability

The datasets used in the current study are available from the corresponding author on reasonable request.

## Abbreviations

AN: Anorexia nervosa;
QoL: Quality of life
ELI: Essen Quality of Life-Index for Eating Disorders
DFlex: Detail and Flexibility Questionnaire
WCST: Wisconsin Card Sorting Test
TMT: Trail Making Test
NT: Navon Task
